# Supplementary material for: MetaRibo-Seq measures translation in microbiomes
Source: Nat Commun. 2020 Jun 29;11:3268. doi: 10.1038/s41467-020-17081-z (PMC7324362; doi:10.1038/s41467-020-17081-z)
Supplement: Supplementary file 10 — Supplementary Data 7 [file 41467_2020_17081_MOESM10_ESM.zip › File2/Confidence_VeryHigh_Taxonomy/981_out.krona.html]

Javascript must be enabled to view this page.

members
magnitude
magnitudeUnassigned
count
unassigned
taxon
rank

981\_out

32

32
superkingdom
2

phylum
544448
8

31969
class
8

8
186329
order

8
2053494
species

SRS011239\_contig\_number\_contig-100\_494.162531SRS014235\_contig\_number\_17133SRS019685\_contig\_number\_41733SRS024009\_contig\_number\_3070SRS048870\_contig\_number\_475SRS054905\_contig\_number\_12696SRS078419\_contig\_number\_contig-100\_764.143919SRS971275\_contig\_number\_25089

3
1239

SRS013098\_contig\_number\_contig-100\_7167.185452SRS016541\_contig\_number\_contig-100\_1938.43063SRS144135\_contig\_number\_27560
24
phylum

species

SRS015782\_contig\_number\_29787
1897034
1

class
91061
16

16
1385
order

16
family
90964

16
genus
1279

1262969

SRS013687\_contig\_number\_31079SRS014979\_contig\_number\_35141SRS016203\_contig\_number\_9014SRS016335\_contig\_number\_6633SRS020233\_contig\_number\_3944SRS022071\_contig\_number\_32000SRS024132\_contig\_number\_42226SRS064757\_contig\_number\_14137SRS065504\_contig\_number\_32909SRS075773\_contig\_number\_6810SRS075984\_contig\_number\_26752SRS104912\_contig\_number\_contig-100\_1980.94782SRS144183\_contig\_number\_contig-100\_548.181588SRS144753\_contig\_number\_contig-100\_9307.55016SRS146812\_contig\_number\_50832SRS147766\_contig\_number\_20039
species
16

class
186801
4

186802
order
4

1

SRS013476\_contig\_number\_17573
species
1898207

3
31979
family

3
1485
genus

species

SRS104400\_contig\_number\_48598
1262788
1

1
species

SRS013965\_contig\_number\_19678
2293052

1
species

SRS147557\_contig\_number\_31488
1896986
